# Supplementary material for: DelGrad: exact event-based gradients for training delays and weights on spiking neuromorphic hardware
Source: Nat Commun. 2025 Sep 9;16:8245. doi: 10.1038/s41467-025-63120-y (PMC12420821; doi:10.1038/s41467-025-63120-y)
Supplement: Supplementary file 1 — Supplementary Information [file 41467_2025_63120_MOESM1_ESM.pdf]

DelGrad: Exact event-based gradients for training delays and weights on spiking neuromorphic hardware

Supplementary Information

SI.A Additional simulation results

SI.A.1 Deeper networks

DelGrad maintains its performance when scaling to deeper networks. Figure SI.1 shows a series of networks including axonal delays with increasing depth (from 1 to 5 hidden layers) and fixed width (7 neurons per hidden layer), demonstrating a consistent decrease in test error. The baseline configuration with 1 hidden layer of 30 neurons falls between the 4-layer and 5-layer networks, both in terms of test error and number of parameters.

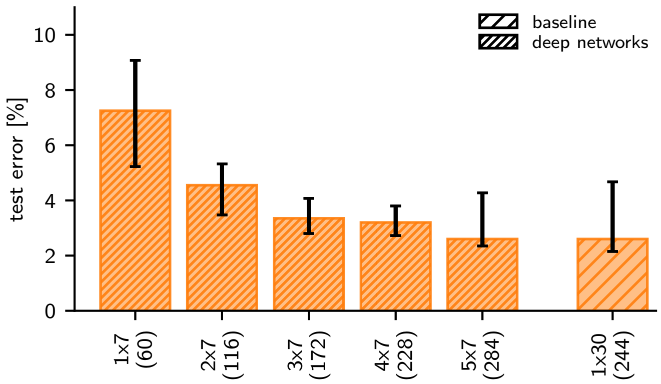

Figure SI.1: **Test error for networks of varying depth.** Networks of the form $l \times n$, with l the number of hidden layers of n neurons. The number of parameters is indicated in parentheses. The test error decreases from a depth $l = 1$ to $l = 5$ and constant width of $n = 7$. The 1×30 baseline lies between the 4×7 and 5×7 configurations.

SI.A.2 Ablation studies

In addition to the results presented in the main text we performed several ablation studies that illustrate the effect of trainable delays in our networks. Figure SI.2b demonstrates that the training makes use of all available resources to improve the task performance by comparing fully trained networks with networks where weight and delay training is disabled either for the input-to-hidden layer connections or for the hidden-to-output ones. The fixed parameters are initialized by sampling from a Gaussian distribution that approximates the empirical distribution of weights and delays observed in a fully trained and optimized baseline network. This ensures that the parameters lie in an appropriate range to solve the task. On the one hand, this shows that the training of all layers is required to obtain optimal performance on the task. On the other hand, it also proves that in the full training, useful gradients are provided to all parameters in all layers.

Finally, we compare the performance achieved when we train weights and delays to an approach similar to the one employed in [1], where weights are trained, but the delays are fixed and random (Fig. SI.3). The mean and standard deviation for configuring the delays are obtained from a hyperparameter search on a wide range of values. We see that for this task, training the delays compared to just providing a random selection that is not adjustable provides a clear advantage for all delay types and especially in smaller networks.

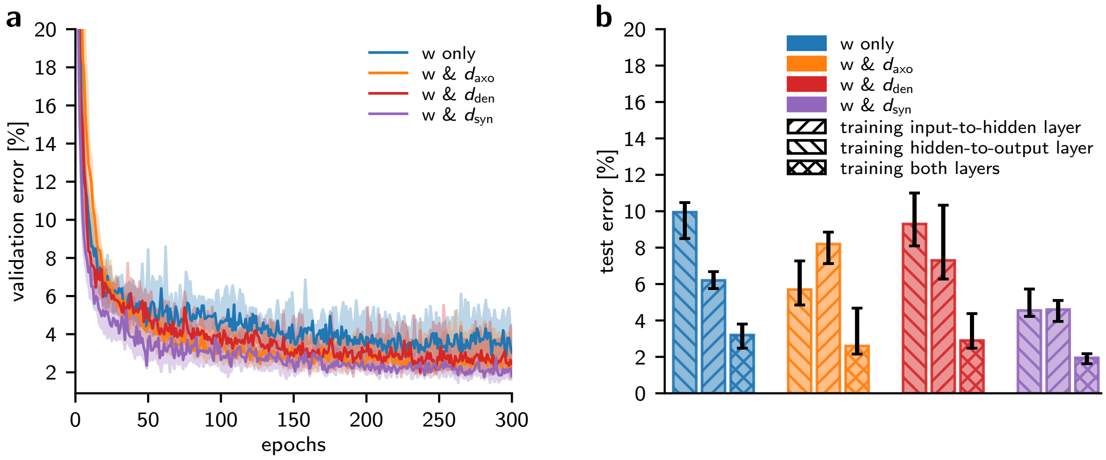

Figure SI.2: **Extended simulation results. a)** Comparison of the validation error during training for the different delay types and a network without delays. All networks have one hidden layer with 30 neurons. **b)** Ablation study (for networks with a hidden layer size of 30 neurons) on the effect of only training the connections between input and hidden layer or between hidden and output layer.

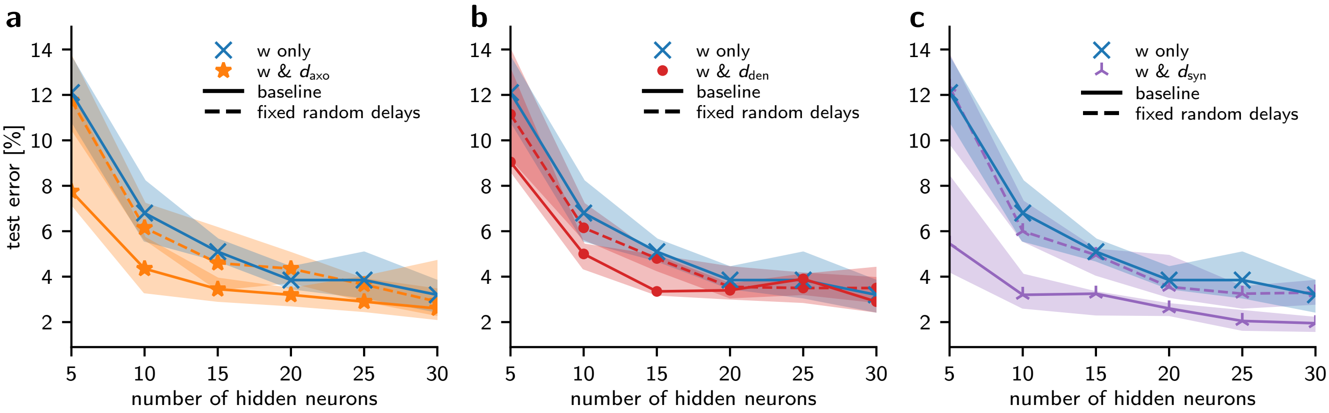

Figure SI.3: **Comparison to random but fixed delays.** For a setup of axonal (a), dendritic (b) or synaptic (c) delays the performance of fully trained networks (i.e. learning weights and delays) are compared to networks with random but fixed delays (dashed lines) and networks without delays (blue).

SI.A.3 Hardware-aware software simulations

The training of spiking neural networks (SNNs) on mixed-signal neuromorphic hardware brings several additional challenges compared to an ideal software simulation. These challenges include, among others, limited resolution and ranges on parameters such as the synaptic weights, fixed-pattern noise and trial-to-trial variability. The impact of these factors on the final outcome of the training difficult to predict and disentangle. To nevertheless get an impression of this, we attempt to mimic these effects in a software simulation. We call this hardware-aware training. With the hardware-aware simulation we can perform an ablation-study that allows us to step-by-step include more levels of hardware-realism and observe the effect on performance. We ensure that the noise levels and restrictions that we include in the simulation are of similar magnitude than what is encountered on a mixed-signal platform. This of course strongly depends on the choice of neuromorphic system and we show it here for our configuration of BSS-2.

Weight ranges and quantization The synaptic weights on BSS-2 have a 6 bit-resolution. To model this in software we use the same approach as described in detail in the methods of [2]: We match the available maximal postsynaptic potential (PSP) height in simulation and on hardware and then train with quantization-aware training within the available range.

Due to the limited weight range, the maximum achievable weight is constrained, requiring, for our chosen neuron

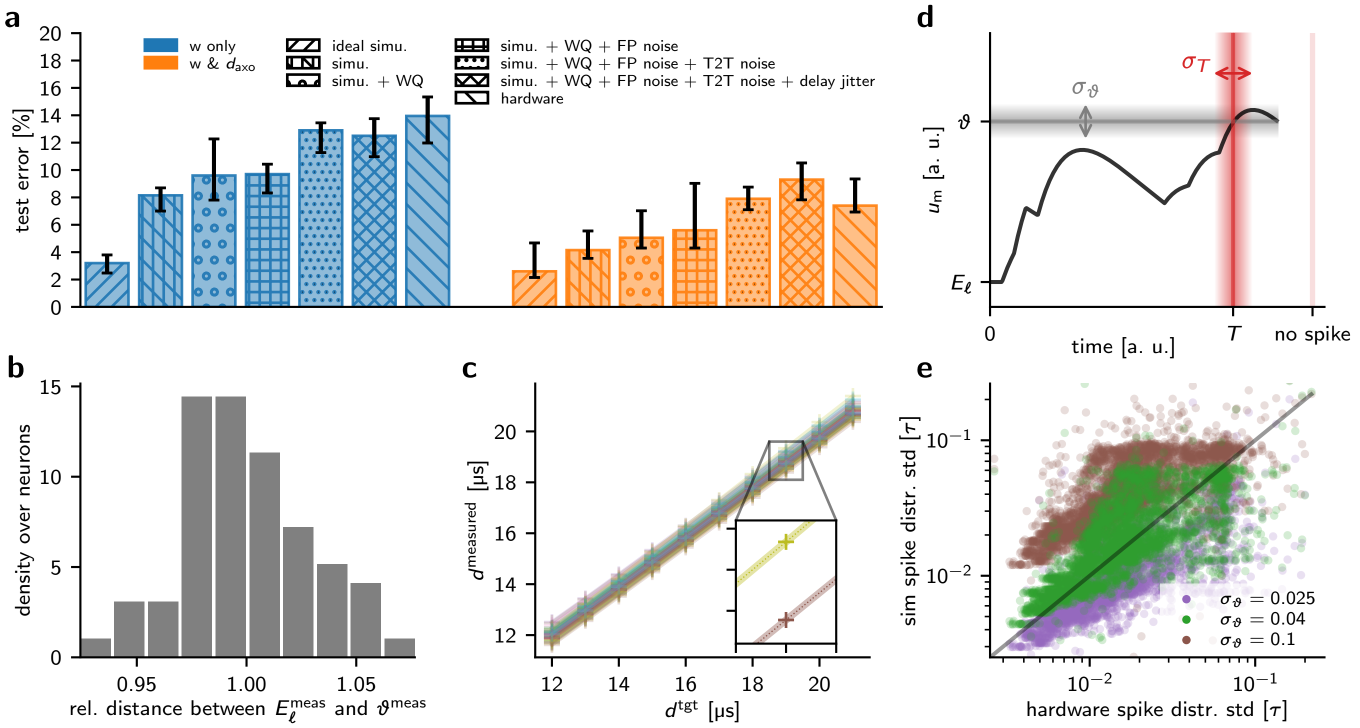

Figure SI.4: **Hardware-aware simulation and noise measurements a)** Ablation study to disentangle the impact of different hardware effects on training performance. In addition to the ideal software simulation (leftmost bar) and the actual hardware result (rightmost bar), it shows hardware-aware simulation results with progressively more hardware effects (from left to right): weight quantization (WQ), fixed-pattern noise (FP), trial-to-trial noise (T2T) and delay jitter. In principle, for each setting slightly different hyperparameters would be optimal; however, for a more direct comparison within reasonable simulation time, we've selected the set of hyperparameters that have proven reliable in the actual hardware emulation for all experiments starting from the second bar (simu.). Bar height indicates median test error and the error bars show the interquartile range (IQR). The values shown correspond to Table SI.1. **b)** Histogram of the normalized relative distance between the measured threshold ϑ and the leak potential E_ℓ. This distribution is used to estimate the fixed-pattern noise between different neuron circuits on the hardware. **c)** Measurements of delays produced by parrot neurons on BrainScaleS-2 (BSS-2) over the corresponding target values used to configure the parrots. **d)** Sketch of the non-linear relationship between variations in the threshold (gray area) and the resulting variations in the output spike timing (red area). **e)** Comparison of variations on the output spike timing on hardware and in the hardware-aware simulation for different assumed trial-to-trial noise magnitudes σ_ϑ. Each point represents the variations on the spike time for one neuron that was presented multiple times with the same input pattern. The plot combines data for multiple neurons and different input patterns.

parametrization, multiple input spikes to reliably trigger an output spike in a neuron. To address this, as described in [2], we replicate each input spike across five channels. This approach effectively increases the maximum achievable weight by a factor of five, a technique we refer to as "channel multiplexing". While these multiplexed channels introduce additional parameters to the network, in the weight-only training they do not enhance its computational capacity. This is because all multiplexed channels share the same input and hence, for each input spike the actual input into the hidden layer is simply the sum of the weights across all channels. In contrast, when delays are available, each channel can have a unique delay, resulting in input spikes arriving at different times. These staggered spike timings separate the influence of each channel, allowing the individual weights to have a distinct effect and thereby increasing the computational capacity of the delay-based network compared to the weight-only ones. However, since this computational advantage arises solely from hardware limitations, we enforce shared delays across multiplexed channels in the delay-based network to ensure a fair comparison.

For the small networks with a hidden layer size of only 5 neurons we encounter the same problem of having too few spiking neurons in the hidden layer to reliably activate the output layer. On BSS-2 we can solve this by using two synapse circuits instead of one for a connection, effectively doubling the weight (the delays here are shared automatically,

because the spikes are received from a parrot neuron). This requires more chip resources per connection, which is why typically this is avoided, but for the smallest networks it proved to be necessary. We mimic this in the hardware-aware simulation, for the hidden layer size of 5, by increasing the available weight range by a factor of 2.

Fixed-pattern noise Fixed-pattern noise, or sometimes called frozen noise, is caused by imperfections in the chip fabrication process and causes slight differences between the neuron circuits. This results in the dynamics of each neuron on chip to differ slightly. These differences between neurons are static over time. The effects of fixed-pattern noise can partially be mitigated using calibration procedures, which we employ, but some variability between the neurons remains. Neuron parameters that are affected by fixed-pattern noise are for example the time constants, the strength of the synaptic input, the resting potential and the threshold. As a simplification for our simulations we do not model all sources of fixed-pattern noise individually, but summarize them all into one source. The variation between neurons of the difference between resting potential and threshold is easy to measure on chip (Fig. SI.4b). Therefore, in our simulations, we model the fixed-pattern noise based on this parameter. In the hardware-aware simulations, at network initialization, a random offset to the threshold of each neuron is drawn from a Gaussian distribution and applied for the whole training procedure. For an estimate on the variance of the Gaussian, we use the variance observed in Fig. SI.4b and increase it slightly to account for the other sources of fixed-pattern noise.

Trial-to-trial variability In addition to fixed-pattern noise, which is static over time, we also observe trial-to-trial variability on the hardware. Electronic circuits are affected by temporal noise on all timescales. High frequency components become apparent as visible jitter on top of the underlying signal, for example on membrane traces. Lower frequency components, in contrast, occur also on timescales often greater than individual observation periods and can thus manifest themselves as pseudo-static offsets of a signal on a trial-to-trial basis. These low frequency components are particularly strong due to the fact that the noise characteristics of electronic devices are typically dominated by flicker noise with a spectral density, or "amplitude", proportional to $1/f$.

The resulting trial-to-trial variability can be interpreted as random fluctuations of neuron parameters on comparatively long time scales: We model it by varying the neuron parameters between experiments (i.e. different batches) but assume them to stay constant within experiments. Most noise sources – such as the leak and threshold terms but also the synaptic integrators and input circuits – eventually affect the neuronal membrane state in form of a random offset and we thus subsume all of them in a variation of the distance between leak and threshold potentials. Therefore, if the same neuron on the same chip is presented with the same input in different batches, its output spike time will differ slightly. The relationship between a variation on the threshold and the resulting variations on the spike times of the neuron is highly non-linear (Fig. SI.4d). This makes the trial-to-trial variability hard to model directly on the spike times, even though this is where it is observed. Instead, we choose to add random offsets to each threshold of every neuron for every batch, which automatically then approximates the trial-to-trial noise observed on the spike times on the hardware. To confirm this, we repeatedly present the same batches of samples to the hardware and record all occurring spikes. Then we present the same samples repeatedly to the hardware-aware simulation, where for each batch and neuron, we add a random sample as the offset to the threshold, drawn from a Gaussian centered around zero and with width σ. For the right choice of σ we observe that the variances, observed for each sample over many repetitions, match well between hardware-aware simulation and experiments (Fig. SI.4e).

Delay effects Figure SI.4c provides an estimate of how accurately our parrot neuron setup reproduces a target delay. While the variations across multiple trials are minimal, we account for them in the hardware-aware simulations. Specifically, we model the variability of the delay circuits by introducing Gaussian noise ($\sigma = 0.01\,\tau$) to the output spike times of the delay layers. In general, spike signal communication on BSS-2 is highly reliable, and spike loss only becomes a concern at very high firing rates within the network. However, the inclusion of parrot neurons for delay implementation introduces a potential new source of spike loss. We measured the average rate of spike loss caused by the parrot neuron setup and found it to be negligibly small. Consequently, this effect is not incorporated into our simulations.

Results With the hardware-aware simulation setup described above we perform an ablation study and compare the results to the actual hardware emulation as well as the ideal software simulation (Fig. SI.4a and Table SI.1).

Some increase in error is introduced when transitioning from the hyperparameters optimized for the ideal simulations to those used on our hardware. This adjustment was necessary because the optimal parameters identified by

software-based Hyperparameter Optimization (HPO) did not perform well on the hardware. In software simulations, hyperparameters were optimized independently for each network size and delay configuration (e.g., axonal or synaptic). However, such extensive HPO is impractical on hardware due to the inability to parallelize runs on the chip, making the process prohibitively time-consuming. To address this, we optimized a single set of hyperparameters that performs reasonably well across both weight-only and axonal delay scenarios and for all network sizes. While this compromise set does not achieve the same performance as the highly optimized software case, it is a good trade-off between the feasibility and performance. More importantly, as shown in Fig. SI.4a and Table SI.1, all modeled noise sources contribute to the increased error, and the final error in hardware-aware training matches closely with the results observed on the chip. This demonstrates that, despite significant simplifications in modeling hardware effects, our measurement-based estimation of noise sources effectively captures the chip's general behavior.

Table SI.1: **Estimation of the impact of different hardware phenomena on training results using the hardware-aware simulation framework.** The values given are the median test errors with the IQR in parentheses.

	ideal simulation	simulation (HW params)	simulation (HW params) + weight quant.	simulation (HW params) + weight quant. + FP noise	simulation (HW params) + weight quant. + FP noise + trial-to-trial	simulation (HW params) + weight quant. + FP noise + trial-to-trial + delay jitter	hardware
weights + axonal delays	$2.60_{2.15}^{4.67}$ %	$4.15_{3.55}^{5.55}$ %	$5.05_{4.30}^{7.03}$ %	$5.60_{4.30}^{9.03}$ %	$7.90_{7.10}^{8.75}$ %	$9.30_{7.83}^{10.52}$ %	$7.40_{6.93}^{9.35}$ %
weights	$3.20_{2.48}^{3.80}$ %	$8.15_{7.00}^{8.70}$ %	$9.60_{7.80}^{12.27}$ %	$9.70_{8.33}^{10.42}$ %	$12.50_{10.97}^{13.75}$ %		$13.95_{11.97}^{15.34}$ %

SI.A.4 Comparison to literature

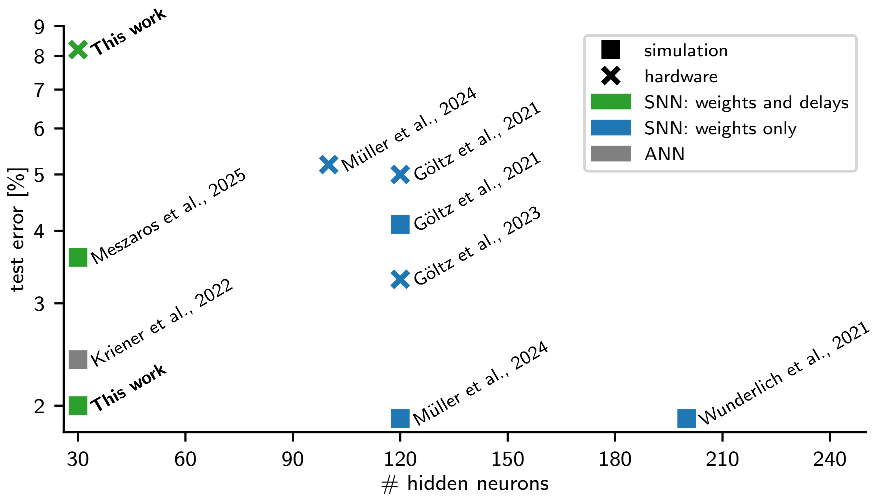

Figure SI.5: **Comparison to other results on the Yin-Yang (YY) dataset.** Each data point represents the mean test accuracy achieved on the YY dataset for a certain size of the hidden layer. Simulation results are marked as squares while hardware results are plotted as crosses. Results employing delays are colored in green, while weight-only networks are blue. The data is collected from the following publications: Meszaros et al., 2025 [3]; Kriener et al., 2022 [4]; Müller et al., 2024 [5]; Göltz et al., 2021 [2]; Göltz et al., 2023 [6] and Wunderlich et al., 2021 [7].

SI.B Hardware implementation and additional results

As BSS-2 does not include dedicated circuitry for emulating delays, we re-purpose neuron circuits to act as delay elements (parrot neurons, see Fig. 5a). For any incoming spike the parrot neuron is configured to produce an output spike with a certain controllable delay, as described below.

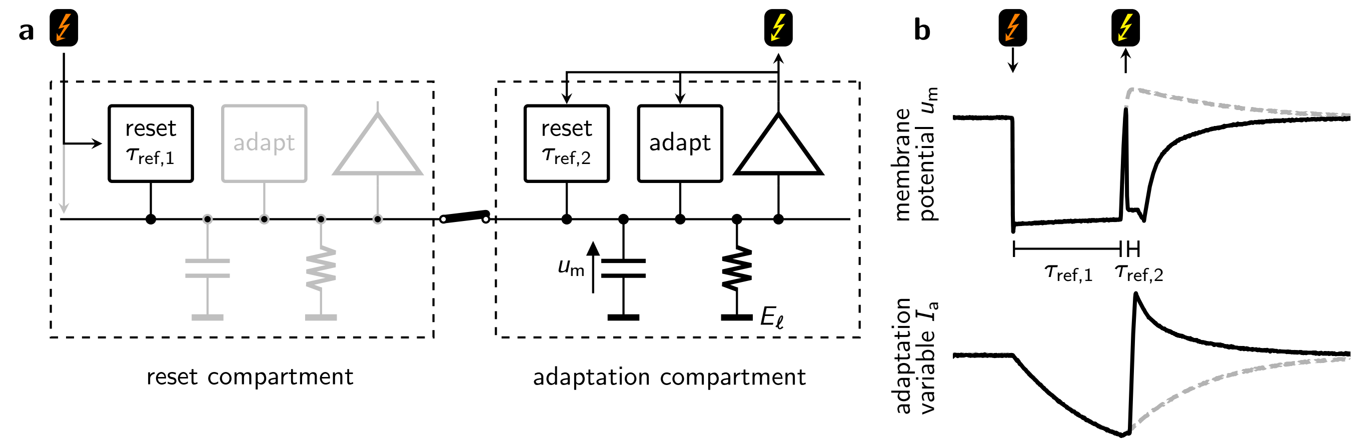

Figure SI.6: **Schematic illustration of the parrot neuron used to implement delays on BrainScaleS-2. a)** Sketch of the two neuron compartments (dashed boxes) used for the delay mechanism. The reset and adaptation circuits are drawn as rectangles, the threshold comparator as triangles. Disabled components are drawn in gray. Lightning bolts indicate incoming (orange) and outgoing (yellow) spikes. **b)** Recorded traces of membrane voltage and adaptation when the parrot neuron receives an input spike and produces a delayed output spike. The dashed gray traces show the circuit with disabled threshold comparator in the second compartment.

SI.B.1 Axonal delays using AdEx and multi-compartment functionality

To obtain the results shown in the main text the delays are implemented using the multi-compartment and the adaptation functionality [8] of the BSS-2 hardware [9, 10]. Figure SI.6 illustrates the mechanism that consists of two separate neuron compartments, from here on called reset and adaptation compartment, corresponding to their respective role. The membrane capacitance of the reset compartment is disabled and the two compartments are short-circuited such that they share a membrane voltage. The dynamics of the adaptation compartment are described by the equations of the adaptive exponential leaky integrate-and-fire (AdEx) model with disabled exponential component:

$$\tau_m \dot{u}_m(t) = [E_\ell - u_m(t)] + I_s(t)/g_\ell - I_a(t)/g_\ell \tag{SI.1}$$

$$\tau_a \dot{I}_a(t) = a(u - E_\ell) - I_a(t) \tag{SI.2}$$

where the adaptation variable I_a is a leaky integrator that is driven by the difference between the membrane voltage and the leak potential. Note that no synaptic input is connected to this compartment, and thus $I_s(t)$ is zero at all times. When $u_m(t)$ crosses the spiking threshold at t_{spike}, the membrane voltage is reset and clamped to V_{reset} for the duration of the refractory period $\tau_{ref,2}$ and spike-triggered adaptation causes a jump on the adaptation variable

$$u(t) = V_{reset} \ \ \forall t \in (t_{spike}, t_{spike} + \tau_{ref,2}] \tag{SI.3}$$

$$I_a \to I_a + b \tag{SI.4}$$

where b is the parameter controlling the magnitude of the spike-triggered adaptation.

An input spike arriving at the reset compartment of the parrot neuron triggers an immediate reset which clamps the membrane potential (shared between both compartments) to the low reset potential V_{reset} for the duration of $\tau_{ref,1}$. Since V_{reset} is lower than E_ℓ, the magnitude of the adaptation current I_a in the adaptation compartment builds up during the refractory period. Once the refractory period $\tau_{ref,1}$ ends, the membrane voltage, now driven by the adaptation variable, can evolve freely away from V_{reset} and the accumulated strong adaptation current I_a causes the membrane voltage to rapidly increase towards the threshold. This phenomenon, although commonly caused not by a low V_{reset} but by inhibitory input, is known as inhibitory rebound: The membrane potential overshoots the leak potential and becomes high enough to reach the spiking threshold of the adaptation compartment and produce an output spike, triggering the reset mechanism of this compartment. Additionally, the spike triggers the spike-triggered adaptation mechanism of the AdEx model, which causes a jump on I_a by b, bringing back the adaptation I_a close to zero. Finally, the refractory period of the adaptation compartment $\tau_{ref,2}$ is configured to be very short and the membrane can almost instantly relax to the leak potential again. After this, the parrot neuron is ready to receive and delay the next input spike.

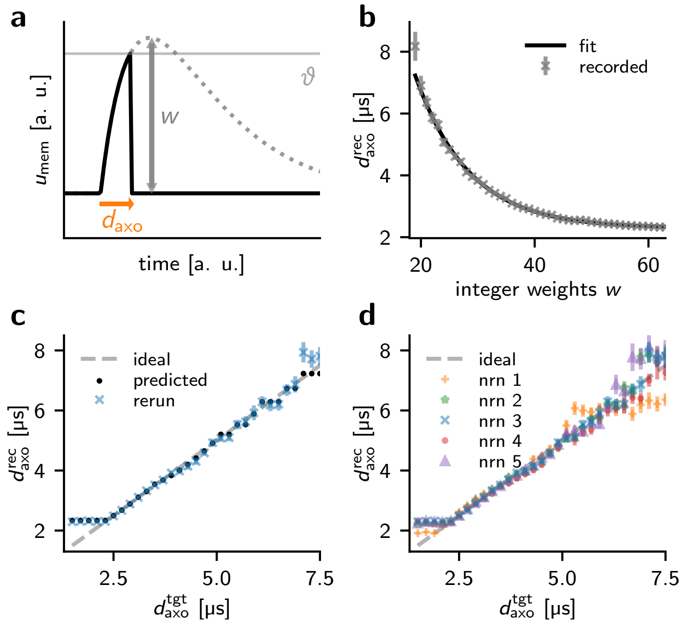

Figure SI.7: **Proof of concept for implementing on-chip axonal delays using only LIF dynamics on BrainScaleS-2.** **a)** Example membrane trace of a parrot neuron where the rise time of the PSP causes a delay of its output spike with respect to the output spike time of its afferent network neuron. This delay can be configured through an appropriate choice of the synaptic weight between network neuron and parrot neuron. **b)** Example BrainScaleS-2 recording of the relationship between the measured input-output delay of a parrot neuron d_{rec} and its afferent synaptic weight (neuron 3 in d)). Mean and standard deviation are shown over 10 runs with 50 spike pairs each. An exponential fit (black) yields the calibration curve for the weight-delay relationship. **c)** Test of the calibration for the same parrot neuron as in b). The calibration curve from the fit in d) is used across a range of target delays $d_{\mathrm{axo}}^{\mathrm{tgt}}$ to determine the corresponding optimal synaptic weights. With this weight the delay is re-measured for 5 runs with 50 spike pairs each, checking the deviation between the predicted delay (black) and the actual recorded delay d_{rec} (mean and standard deviation in blue). **d)** Same as c) but for 5 different parrot neurons on the chip to illustrate the variability between different neuron circuits.

As the inhibitory rebound causes an output spike immediately after the end of the refractory period of the reset compartment, the delay of this parrot neuron can be configured via $\tau_{\mathrm{ref,1}}$. This is advantageous, as τ_{ref} is an 8 bit digital parameter on BSS-2 and can be configured to a large range of possible refractory periods without fixed pattern noise. As shown in Fig. SI.4c, the delays can be reliably configured in the range of 12 μs to 21 μs. In principle, the available delay range is larger (approximately 8 μs to 35 μs), but since we do not require such a large range of delays for our experiments and the values in the middle of the available range are the most stable, we restrict ourselves to what is shown in Fig. SI.4c. Note that having the smallest available delay unequal to zero is not computationally relevant, as long as this minimal value is equal for all neurons.

SI.B.2 Proof-of-concept for axonal delays using only LIF dynamics

Since leaky integrate-and-fire (LIF) neuron models are more widely available on various neuromorphic platforms compared to the multi-compartment AdEx model, we also present a proof-of-concept of how LIF models can be adapted for implementing analog on-chip delays, ensuring our results are more easily reproducible.

Setup The network setup in this method is similar to the previous section in that each network neuron has a corresponding parrot implementing the delay; However, the method of creating this delay is different: We leverage the fact that due to the finite rise time of the PSP on the parrot's membrane voltage, its spike is delayed compared to the one of the network neuron (Fig. SI.7a). The magnitude of this delay, which emulates the axonal delay of the network neuron, depends on several parameters, such as the synaptic weight w of the connection between the network and parrot neurons, the time constants $\tau_{\mathrm{s}}, \tau_{\mathrm{m}}$ and the difference between threshold and leak potential of the parrot neuron. For a theoretical description of the relationship between the parrot's delay and the synaptic weight see Section SI.F.

For our implementation of this scheme on BSS-2, we control the delay solely via the synaptic weight w, keeping the time constants and potentials fixed: while those parameters can be individually tuned, that (analog) configuration is slower compared to the (digital) weight setting. Since in our trained networks on BSS-2 ([2] and Fig. 5) we use neuron time constants of $\tau_\mathrm{s} = \tau_\mathrm{m} \approx 6\,\mu\mathrm{s}$, we aim to reach delays of the same magnitude here. To achieve this, we configure the parrot neurons to have a synaptic time constant of $\tau_\mathrm{s} = 10\,\mu\mathrm{s}$ and a membrane time constant of $\tau_\mathrm{m} = 15\,\mu\mathrm{s}$. A long refractory time of $16\,\mu\mathrm{s}$ ensures that each input to the parrot only triggers one output spike.

During the training of a network, it is required to reconfigure the parrot neurons on the chip to produce the correct axonal delays $d_\mathrm{axo}^\mathrm{tgt}$. For this, the relationship between the synaptic weight w and the produced delay $d_\mathrm{axo}^\mathrm{rec}$ needs to be measured. Due to the usual variations in the manufacturing process (fixed-pattern noise), the on-chip analog neuron circuits are not exactly identical to each other. Therefore, for every parrot neuron, we perform a separate calibration measurement in order to determine the precise mapping between the weight parameter and the recorded delay individually. To this end, we configure a range of different weights and record the resulting delays $d_\mathrm{axo}^\mathrm{rec}$ (Fig. SI.7b for an example neuron). The full available weight range from 0 to 63 is not used, as for the lower weights, the parrot neuron does not reliably produce output spikes. To include both temporal drift during one trial and trial-to-trial variations, we record delays during 10 runs with 50 pairs of input and output spikes and average the results.

We fit an exponential function $d(w) = \alpha + \beta \exp(\gamma(w + \delta))$ to the measured data. The inverse of the fit function thus determines the relation between the target delay $d_\mathrm{axo}^\mathrm{tgt}$, and the optimal integer weight to configure on the chip. To test the quality of the weight-delay fit, it is used to configure the chip for a whole range of target delays $d_\mathrm{axo}^\mathrm{tgt}$, while measuring the actual value of the delays produced on the chip $d_\mathrm{axo}^\mathrm{rec}$ (Fig. SI.7c). The above process is repeated for 5 different neuron circuits on the chip, and the results are compared to illustrate the impact of fixed-pattern noise in Fig. SI.7d.

Results Figure SI.7c shows the desired correspondence between the target, $d_\mathrm{axo}^\mathrm{tgt}$, and the recorded, $d_\mathrm{axo}^\mathrm{rec}$, on-chip delay values, especially in the intermediate delay range. This underpins the feasibility of our proposed approach. We note a plateau in the recorded delay values for lower targets, caused by the maximum possible on-chip weight value, corresponding to the shortest possible delay. Additionally, a larger deviation and more instability is observed for larger target delays; this effect has two causes: a worse quality of the exponential fit and an increased instability of the threshold crossing in the region where the PSP plateaus. Such increasing instability is unavoidable in analog neurons, as any amount of noise on the membrane voltage results in increased trial-to-trial variability when the peak of the PSP is close to the threshold.

Although each neuron can be configured individually to produce the desired behavior, there is still some variability between the neurons. However, we do not expect these variations to be harmful in practice; in fact, some heterogeneity between neurons behavior might even be beneficial, as has been shown in [11].

While the presented idea can be feasible for small networks and tasks, it is clearly suboptimal, as it requires a portion of the available neuron circuits to be used as delay elements instead of their usual role in the network. Additionally, several practical considerations have to be taken into account when this setup is included in the training of a full network. First, the range of achievable delays is limited by the time constants of the parrot neurons. In our experiments we targeted a delay range of approximately $6\,\mu\mathrm{s}$ which corresponds to the delay range used in the simulation results. Second, for a correct delay on an incoming spike, the parrot neuron's membrane voltage and synaptic currents need to be at their resting values. Therefore, the interval between spikes arriving at the parrot neuron needs to be large enough, which can be ensured by increasing the refractory time of the neurons in the network. However, for tasks where the neurons need short refractory periods to process their input correctly, this method of producing axonal delays is not suitable. Nevertheless, these results provide a proof of concept that axonal delays can be implemented by repurposing resources and circuits that are universally available on most neuromorphic substrate.

SI.C Complete equation for the time of the first spike

Given a sequence of input spikes $\{t_i\}$ and corresponding weights $\{w_i\}$, we define

$$a_n := \sum_{i \in C} w_i \exp\left(\frac{t_i}{n\tau_\mathrm{s}}\right) \quad \text{and} \quad b := \sum_{i \in C} w_i \frac{t_i}{\tau_\mathrm{s}} \exp\left(\frac{t_i}{\tau_\mathrm{s}}\right) . \tag{SI.5}$$

These definitions use the causal set $C = \{i \mid t_i < T\}$ of input spikes before the output. With those definitions, the spike time of a neuron with identical membrane and synaptic time constant $\tau_\mathrm{m} = \tau_\mathrm{s}$ is (Eq. (4) in the main text)

$$T = \tau_\mathrm{s} \left\{ \frac{b}{a_1} - \mathcal{W}\left[-\frac{g_\ell \vartheta}{a_1} \exp\left(\frac{b}{a_1} \right) \right] \right\} , \tag{SI.6}$$

and for $\tau_\mathrm{m} = 2\tau_\mathrm{s}$ (Eq. (5) in the main text)

$$T = 2\tau_\mathrm{s} \ln \left[\frac{2a_1}{a_2 + \sqrt{a_2^2 - 4a_1 g_\ell \vartheta}} \right] . \tag{SI.7}$$

The Lambert W function is defined as the solution $h = \mathcal{W}(z)$ to the equation $z = h \exp(h)$.

SI.D Extending the formalism for multiple spikes

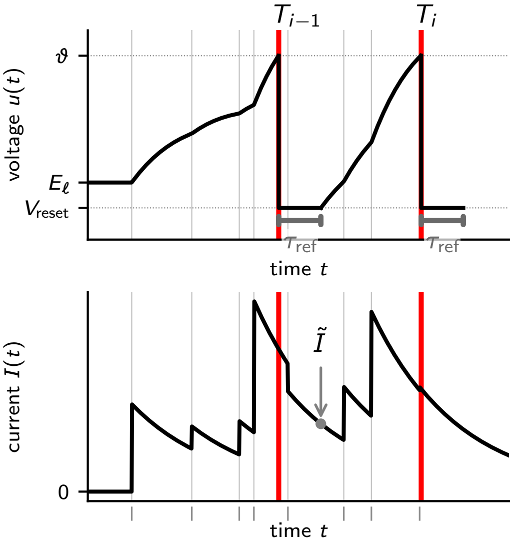

Figure SI.8: **Sketch of the voltage (top) and current (bottom) dynamics of an LIF neuron that is spiking multiple times.** The gray vertical lines indicate the input spikes, the red vertical lines the output spikes. After each spike, the voltage is clamped to the reset potential V_reset for a time τ_ref, highlighted by the gray horizontal bar in the top plot. After this refractory period, the voltage evolves freely again, from $T_{i-1} + \tau_\mathrm{ref}$ on driven by the residual current $\tilde{I}$ and additional input spikes.

In the main text, we have derived our equations in a simplified scenario in which each neuron only spikes once. As shown in the present and earlier work [2], single spikes can already be sufficient for many problems, however, for scenarios in which multiple spikes per neuron are necessary, the extended equations are derived below.

For the first spike of a neuron, Equations (4) and (5) are derived by integrating the ordinary differential equation (ODE) in Eq. (1) to get voltage dynamics $u(t)$, and afterward solving for the time T of the spike, defined by $u(T) = \vartheta$. Recalling the dynamics of the LIF model, after a spike the voltage is fixed at the reset voltage V_reset for a time τ_ref (Fig. SI.8, top panel), and afterward continues to follow the dynamics laid out in the ODE (1). For the time of the second spike T_2 and all further spikes this implies that the same procedure can be followed when adhering to different initial conditions.

For the first spike, the integration of the ODE is performed with initial vanishing current $I(0) = 0$ and voltage $u(0) = 0$ (w.l.o.g. we have chosen leakage $E_\ell = 0$), resulting in voltage dynamics

$$u(t) = \sum_{i \in C} \Theta(t - t_i) \frac{w_i}{g_\ell} \frac{\tau_\mathrm{s}}{\tau_\mathrm{m} - \tau_\mathrm{s}} \left[\exp\left(-\frac{t - t_i}{\tau_\mathrm{m}} \right) - \exp\left(-\frac{t - t_i}{\tau_\mathrm{s}} \right) \right] . \tag{SI.8}$$

Assuming a spike at T_{i-1}, the new initial condition for the voltage can be written down at time $\tilde{t} := T_{i-1} + \tau_\mathrm{ref}$ as

$$u(\tilde{t}) = \tilde{u} := V_\mathrm{reset} , \tag{SI.9}$$

while the current is not affected by the reset, and keeps following Eq. (2). To simplify the notation, we split up the spikes of the causal set $t_i \in C$ into one set arriving before $\tilde{t}$, $C^< = \{t_i \in C | t_i < \tilde{t}\}$, and one set with spikes afterward $C^\geq = \{t_i \in C | t_i \geq \tilde{t}\}$. For times $t \geq \tilde{t}$, we can write the current as

$$I(t) = \tilde{I} \exp\left(-\frac{t - \tilde{t}}{\tau_\mathrm{s}} \right) + \sum_{i \in C^\geq} \Theta(t - t_i) w_i \exp\left(-\frac{t - t_i}{\tau_\mathrm{s}} \right) , \tag{SI.10}$$

with $\tilde{I} = \sum_{i \in C^<} w_i \exp(-\frac{\tilde{t} - t_i}{\tau_\mathrm{s}})$ the (residual) current at time $\tilde{t}$ (see Fig. SI.8). Integration of the ODE under these initial conditions results in an equation for the dynamics of the voltage:

$$\begin{aligned} u(t) = &\sum_{i \in C^\geq} \Theta(t - t_i) \frac{w_i}{g_\ell} \frac{\tau_\mathrm{s}}{\tau_\mathrm{m} - \tau_\mathrm{s}} \left[\exp\left(-\frac{t - t_i}{\tau_\mathrm{m}} \right) - \exp\left(-\frac{t - t_i}{\tau_\mathrm{s}} \right) \right] \\ &+ \tilde{u} \exp\left(-\frac{t - \tilde{t}}{\tau_\mathrm{m}} \right) + \frac{\tilde{I}}{g_\ell} \frac{\tau_\mathrm{s}}{\tau_\mathrm{m} - \tau_\mathrm{s}} \left[\exp\left(-\frac{t - \tilde{t}}{\tau_\mathrm{m}} \right) - \exp\left(-\frac{t - \tilde{t}}{\tau_\mathrm{s}} \right) \right] . \end{aligned} \tag{SI.11}$$

The first term encapsulates the effect of the incoming spikes as before (compare Eq. (SI.8)), while the second line is an exponential decay from the reset to the leak, while accounting for the effect of the residual current. Interestingly, this form of the equation shows that the residual current can be modeled as a virtual spike with weight $\tilde{I}$ at time $\tilde{t}$. Crucially, when starting at leak voltage $\tilde{u} = 0$ and without initial current $\tilde{I} = 0$, the above voltage dynamics Eq. (SI.8) are recovered.

With this equation, the derivation performed in [2] can be followed, i.e., assuming a spike $T_i > \tilde{t}$ defines a causal set $\tilde{C} = \{t_i \in C^{\geq} | t_i < T_i\}$, and using $u(T_i) = \vartheta$ we can write

$$\begin{aligned} 0 = &- \underbrace{\left[\tilde{I} \frac{\tau_\mathrm{s}}{\tau_\mathrm{m} - \tau_\mathrm{s}} \exp\left(\frac{\tilde{t}}{\tau_\mathrm{s}}\right) + \sum_{i \in \tilde{C}} w_i \frac{\tau_\mathrm{s}}{\tau_\mathrm{m} - \tau_\mathrm{s}} \exp\left(\frac{t_i}{\tau_\mathrm{s}}\right) \right]}_{a_1'} \cdot \exp\left(-\frac{T_i}{\tau_\mathrm{s}}\right) \\ &+ \underbrace{\left[\tilde{u} \exp\left(\frac{\tilde{t}}{\tau_\mathrm{m}}\right) + \tilde{I} \frac{\tau_\mathrm{s}}{\tau_\mathrm{m} - \tau_\mathrm{s}} \exp\left(\frac{\tilde{t}}{\tau_\mathrm{m}}\right) + \sum_{i \in \tilde{C}} w_i \frac{\tau_\mathrm{s}}{\tau_\mathrm{m} - \tau_\mathrm{s}} \exp\left(\frac{t_i}{\tau_\mathrm{m}}\right) \right]}_{a_2'} \cdot \exp\left(-\frac{T_i}{\tau_\mathrm{m}}\right) \\ &- g_\ell \vartheta \\ = &- a_1' \exp\left(-\frac{T_i}{\tau_\mathrm{s}}\right) + a_2' \exp\left(-\frac{T_i}{\tau_\mathrm{m}}\right) - g_\ell \vartheta \; . \end{aligned} \tag{SI.12}$$

This equation follows the same structure as in the original derivation [2, Eq. (26-27)] with differently defined parameters a_1' and a_2': comparing with Eq. (SI.5), in the case of $\tau_\mathrm{m} = 2\tau_\mathrm{s}$ we find

$$\begin{aligned} a_1 \to a_1' &:= a_1 + \tilde{I} \exp\left(\frac{\tilde{t}}{\tau_\mathrm{s}}\right) \\ a_2 \to a_2' &:= a_2 + \tilde{I} \exp\left(\frac{\tilde{t}}{\tau_\mathrm{m}}\right) + \tilde{u} \exp\left(\frac{\tilde{t}}{\tau_\mathrm{m}}\right) \; . \end{aligned} \tag{SI.13}$$

Notably, this implies that one can solve for T_i to get a function

$$T_i \left(\{t_i\} \cup \{w_i\}, \tilde{I}, \tilde{t} \right) \; . \tag{SI.14}$$

This function is at the heart of implementing exact, event-based forward dynamics and, more importantly, its differentiability enables error backpropagation through multiple layers of such neurons. Through $\tilde{I}$ there is a dependence of the output spike times T_i on earlier input spike times $t_i < \tilde{t}$, and through $\tilde{t}$ a dependence on the previous spike of the same neuron. For the case of $\tau_\mathrm{m} = \tau_\mathrm{s}$, one can use l'Hôpital's rule and the Lambert W function to write down the solution for T_i.

SI.E Explicit, event-based gradients of a voltage-max-over-time loss

Typically, when using an event-based framework, information in a network is encoded in spike times. For some tasks, losses based on the voltage of (a subset) of neurons have been proposed and used, especially the max-over-time loss (for a selection, see [6, 7, 12–14]). For this, the corresponding loss function depends on the correct class $n^\star$ and the voltage $\mathbf{u}(t)$ of the (non-spiking) label neurons together with a scale factor a_scale like

$$\mathcal{L}_{\mathrm{MOT}}[\mathbf{u}(t), n^\star; a_\mathrm{scale}] = -\log \left[\mathrm{softmax}_{n^\star}(a_\mathrm{scale} \cdot \max_t \mathbf{u}(t)) \right] \; . \tag{SI.15}$$

Because this loss has been predominantly used with surrogate gradients and depends on the membrane voltage of the label neurons, it is not typically associated with exact and event-based training schemes, with [7, 14] being the exceptions. However, the loss is compatible with a purely spike-based formulation and in the following the relevant gradient will be derived:

$$\frac{\partial u_\mathrm{max}}{\partial \theta} = \frac{\partial u(t|\{\theta\})}{\partial \theta}\Big|_{t=\tilde{t}} + \frac{\partial u(t|\{\theta\})}{\partial t}\Big|_{t=\tilde{t}} \cdot \frac{\partial \tilde{t}}{\partial \theta} \; . \tag{SI.16}$$

In addition to the natural first term, the second term occurs when an (inhibitory) input spike determines the maximum of the voltage.

For the derivation, we assume the voltage u is a function of the parameters input spikes $\{t_i\}$ and weights $\{w_i\}$, here shortened as $\{\theta\}$

$$u = u(t|\{t_i\} \cup \{w_i\}) = u(t|\{\theta\}) \;, \tag{SI.17}$$

the maximum voltage $u_{\max}$ at time $\tilde{t}$ is defined by

$$\begin{aligned} \tilde{t} := &\quad \underset{t\in S}{\arg\max} \quad u(t|\{\theta\}) \\ u_{\max} = &\quad \max_{t\in S} \quad u(t|\{\theta\}) = u(\tilde{t}|\{\theta\}) \;, \end{aligned} \tag{SI.18}$$

where $t \in S$ runs in the integration domain S. This definition makes $u_{\max}$ an implicit function[1] of the parameters $\{\theta\}$. Because the voltage of these (non-spiking) neurons is continuous, the maximization can be written in an integral form using the Dirac δ distribution

$$u_{\max} = \max_t u(t|\{\theta\}) = u(\tilde{t}|\{\theta\}) \tag{SI.19}$$

$$= \int_S \mathrm{d}t\, u(t|\{\theta\})\delta(t-\tilde{t}) \;, \tag{SI.20}$$

which will allow the reformulation in simple, event-based terms.

We split up the voltage along the spike times t_i, cf. Fig. SI.9. Here, w.l.o.g. we assume the input spikes $\{t_i | i \in [1, N]\}$ to be ordered $t_i < t_{i+1} \forall i$ and define $t_0 = -\infty$ and $t_{N+1} = \infty$ as well as the intervals $S_i = (t_i, t_{i+1}]$. Further, we want to define the shorthand $\tilde{S}$ for the interval $\tilde{\imath}$ that contains the maximum $\tilde{S} := S_{\tilde{\imath}} \ni \tilde{t}$, therefore $t_{\tilde{\imath}}$ is the last input spike causal to the dynamics in this interval and there is no new spike within the interval. Employing as a shorthand the synaptic interaction kernel

$$\kappa(t) = \Theta(t) \frac{\tau_\mathrm{s}}{\tau_\mathrm{m} - \tau_\mathrm{s}} \left[\exp(-t/\tau_\mathrm{m}) - \exp(-t/\tau_\mathrm{s})\right] \;, \tag{SI.21}$$

the voltage behaves like $u(t|\{\theta\}) = \frac{1}{g_\ell} \sum_i^N w_i \kappa(t - t_i)$, and one can write

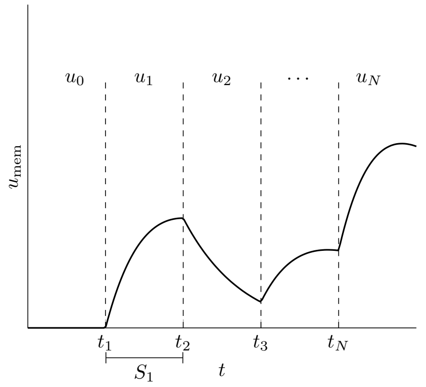

Figure SI.9: Separating u into distinct, smooth u_i at the input spike times t_i.

$$u(t|\{\theta\}) = \frac{1}{g_\ell} \cdot \begin{cases} 0 & \text{if } t \le t_1 \\ w_1\kappa(t-t_1) & \text{if } t_1 < t \le t_2 \\ w_1\kappa(t-t_1) + w_2\kappa(t-t_2) & \text{if } t_2 < t \le t_3 \\ \quad\vdots & \quad\vdots \\ u_n(t|\{\theta\}) & \text{if } t_n < t \le t_{n+1} \end{cases} \;, \tag{SI.22}$$

with $u_n(t|\{\theta\}) = \frac{1}{g_\ell}\sum_i^n w_i\kappa(t-t_i)$. This function $u_n(t)$ is time-differentiable everywhere on its domain S_n. The separation into u_n can be carried over to all integrals of the voltage with any function f

$$\int_S \mathrm{d}t\, u(t|\{\theta\}) \big[f(t)\big] = \sum_i \int_{S_i} \mathrm{d}t\, u_i(t|\{\theta\}) \big[f(t)\big] \;. \tag{SI.23}$$

Specifically, this can be done for the integral formulation of $u_{\max}$ Eq. (SI.20). For the next step, a requirement is the derivative of an integral with varying boundaries:

$$\frac{\partial}{\partial y} \int_{a(y)}^{b(y)} \mathrm{d}x\, f(x,y) = \left[f(x,y) \frac{\partial}{\partial y} x \right]_{x=a(y)}^{x=b(y)} + \int_{a(y)}^{b(y)} \mathrm{d}x\, \frac{\partial}{\partial y} f(x,y) \,. \tag{SI.24}$$

[1] In this section, we assume the maximum is uniquely defined in a neighborhood of the current parameters, i.e., there is no sudden jump of the maximum to another time. Cases in which this assumption is not satisfied have to be treated differently, e.g., by adding up gradients coming from these different maxima of equal value.

Differentiating the integral form of the maximum voltage leads to:

$$\frac{\partial u_{\max}}{\partial\theta} = \frac{\partial}{\partial\theta}\int_S \mathrm{d}t\, u(t|\{\theta\})\cdot\delta(t-\tilde{t}) \tag{SI.25}$$

$$= \frac{\partial}{\partial\theta}\sum_i \underbrace{\int_{S_i} \mathrm{d}t\, u_i(t|\{\theta\})\cdot\delta(t-\tilde{t})}_{\text{vanishes due to }\delta\text{ except if }i=\tilde{\imath}} \tag{SI.26}$$

$$= \frac{\partial}{\partial\theta}\int_{t_{\tilde{\imath}}}^{t_{\tilde{\imath}+1}} \mathrm{d}t\, u_{\tilde{\imath}}(t|\{\theta\})\cdot\delta(t-\tilde{t}) \tag{SI.27}$$

$$= \left[u_{\tilde{\imath}}(t|\{\theta\})\cdot\delta(t-\tilde{t})\frac{\partial t}{\partial\theta}\right]_{t_{\tilde{\imath}}}^{t_{\tilde{\imath}+1}} + \int_{t_{\tilde{\imath}}}^{t_{\tilde{\imath}+1}} \mathrm{d}t\, \frac{\partial}{\partial\theta}\left[u_{\tilde{\imath}}(t|\{\theta\})\cdot\delta(t-\tilde{t})\right] \tag{SI.28}$$

$$= \left[u_{\tilde{\imath}}(t|\{\theta\})\cdot\delta(t-\tilde{t})\frac{\partial t}{\partial\theta}\right]_{t_{\tilde{\imath}}}^{t_{\tilde{\imath}+1}} + \int_{t_{\tilde{\imath}}}^{t_{\tilde{\imath}+1}} \mathrm{d}t\, \left[\frac{\partial u_{\tilde{\imath}}(t|\{\theta\})}{\partial\theta}\cdot\delta(t-\tilde{t}) + u_{\tilde{\imath}}(t|\{\theta\})\cdot\frac{\partial}{\partial\theta}\delta(t-\tilde{t})\right] \tag{SI.29}$$

$$= \left[u_{\tilde{\imath}}(t|\{\theta\})\cdot\delta(t-\tilde{t})\frac{\partial t}{\partial\theta}\right]_{t_{\tilde{\imath}}}^{t_{\tilde{\imath}+1}} + \frac{\partial u(t|\{\theta\})}{\partial\theta}\Big|_{t=\tilde{t}} + \int_{t_{\tilde{\imath}}}^{t_{\tilde{\imath}+1}} \mathrm{d}t\, u_{\tilde{\imath}}(t|\{\theta\})\cdot\frac{\partial}{\partial\theta}\delta(t-\tilde{t})\,. \tag{SI.30}$$

For the last term, the derivative of the delta distribution $\frac{\partial}{\partial\theta}\delta(t-\tilde{t})$ can be reformulated:

$$\frac{\partial\delta(t-\tilde{t})}{\partial\theta} = \frac{\partial\delta(t-\tilde{t})}{\partial(t-\tilde{t})}\frac{\partial(t-\tilde{t})}{\partial\theta} \tag{SI.31}$$

$$= -\frac{\partial\delta(t-\tilde{t})}{\partial(t-\tilde{t})}\frac{\partial\tilde{t}}{\partial\theta} \tag{SI.32}$$

$$= -\frac{\partial\delta(t-\tilde{t})}{\partial(t-\tilde{t})}\overbrace{\frac{\partial(t-\tilde{t})}{\partial t}}^{\text{inserting 1}}\frac{\partial\tilde{t}}{\partial\theta} \tag{SI.33}$$

$$= -\frac{\partial\delta(t-\tilde{t})}{\partial t}\frac{\partial\tilde{t}}{\partial\theta}\,. \tag{SI.34}$$

Inserting this above in Eq. (SI.30) and integrating by parts[2] allows removing the derivative of the δ distribution:

$$\int_{t_{\tilde{\imath}}}^{t_{\tilde{\imath}+1}} \mathrm{d}t\, u_{\tilde{\imath}}(t|\{\theta\})\cdot\frac{\partial}{\partial\theta}\delta(t-\tilde{t}) = -\int_{t_{\tilde{\imath}}}^{t_{\tilde{\imath}+1}} \mathrm{d}t\, u_{\tilde{\imath}}(t|\{\theta\})\frac{\partial\delta(t-\tilde{t})}{\partial t}\frac{\partial\tilde{t}}{\partial\theta} \tag{SI.35}$$

$$= -\left[u_{\tilde{\imath}}(t|\{\theta\})\delta(t-\tilde{t})\frac{\partial\tilde{t}}{\partial\theta}\right]_{t_{\tilde{\imath}}}^{t_{\tilde{\imath}+1}} + \int_{t_{\tilde{\imath}}}^{t_{\tilde{\imath}+1}} \mathrm{d}t\, \delta(t-\tilde{t})\frac{\partial}{\partial t}\left(u_{\tilde{\imath}}(t|\{\theta\})\cdot\frac{\partial\tilde{t}}{\partial\theta}\right) \tag{SI.36}$$

$$= -\left[u_{\tilde{\imath}}(t|\{\theta\})\delta(t-\tilde{t})\frac{\partial\tilde{t}}{\partial\theta}\right]_{t_{\tilde{\imath}}}^{t_{\tilde{\imath}+1}} + \int_{t_{\tilde{\imath}}}^{t_{\tilde{\imath}+1}} \mathrm{d}t\, \delta(t-\tilde{t})\cdot\frac{\partial u_{\tilde{\imath}}(t|\{\theta\})}{\partial t}\cdot\frac{\partial\tilde{t}}{\partial\theta} \tag{SI.37}$$

$$= -\left[u_{\tilde{\imath}}(t|\{\theta\})\delta(t-\tilde{t})\frac{\partial\tilde{t}}{\partial\theta}\right]_{t_{\tilde{\imath}}}^{t_{\tilde{\imath}+1}} + \frac{\partial u_{\tilde{\imath}}(t|\{\theta\})}{\partial t}\Big|_{t=\tilde{t}}\cdot\frac{\partial\tilde{t}}{\partial\theta} \tag{SI.38}$$

$$= -\left[u_{\tilde{\imath}}(t|\{\theta\})\delta(t-\tilde{t})\frac{\partial\tilde{t}}{\partial\theta}\right]_{t_{\tilde{\imath}}}^{t_{\tilde{\imath}+1}} + \frac{\partial u(t|\{\theta\})}{\partial t}\Big|_{t=\tilde{t}}\cdot\frac{\partial\tilde{t}}{\partial\theta}\,. \tag{SI.39}$$

The time derivative only acts on $u_{\tilde{\imath}}$ because $\tilde{t}$ (and its derivative) are independent of the integration variable t. Furthermore, at time $\tilde{t}$ the value of $u_{\tilde{\imath}}$ is identical to the one of u, so we can substitute the regular voltage back in.

With Eq. (SI.39) inserted into Eq. (SI.30), reordering of the terms yields

$$\frac{\partial u_{\max}}{\partial\theta} = \left[u(t|\{\theta\})\cdot\delta(t-\tilde{t})\frac{\partial t}{\partial\theta}\right]_{t_{\tilde{\imath}}}^{t_{\tilde{\imath}+1}} - \left[u(t|\{\theta\})\cdot\delta(t-\tilde{t})\frac{\partial\tilde{t}}{\partial\theta}\right]_{t_{\tilde{\imath}}}^{t_{\tilde{\imath}+1}} + \frac{\partial u(t|\{\theta\})}{\partial\theta}\Big|_{t=\tilde{t}} + \frac{\partial u(t|\{\theta\})}{\partial t}\Big|_{t=\tilde{t}}\frac{\partial\tilde{t}}{\partial\theta}\,. \tag{SI.40}$$

[2] $\int \mathrm{d}x\, f(x)\frac{\partial g(x)}{\partial x} = f(x)g(x)\big|_{\text{boundary}} - \int \mathrm{d}x\, \frac{\partial f(x)}{\partial x}g(x)$

From $\tilde{t} \in S_{\tilde{\imath}} = (t_{\tilde{\imath}}, t_{\tilde{\imath}+1}]$ follows $\tilde{t} \neq t_{\tilde{\imath}}$, therefore the first two terms evaluated at the lower boundary vanish. The remaining, upper boundary terms $t = t_{\tilde{\imath}+1}$ are only nonzero if the maximum happens at that boundary $\tilde{t} = t_{\tilde{\imath}+1}$, in which case the two terms cancel each other, yielding the final, concise result

$$\frac{\partial u_{\max}}{\partial \theta} = \frac{\partial u(t|\{\theta\})}{\partial \theta}\Big|_{t=\tilde{t}} + \frac{\partial u(t|\{\theta\})}{\partial t}\Big|_{t=\tilde{t}} \cdot \frac{\partial \tilde{t}}{\partial \theta} \,. \tag{SI.41}$$

There are two distinct cases how a maximum of the voltage can be reached: The more common one is a maximum due to the decay of the voltage back to the leakage. In a neighborhood around this maximum at $\tilde{t}$, the voltage is a smooth function with time derivative $\frac{\partial u}{\partial t}|_{t=\tilde{t}} = \dot{u}(\tilde{t}) = 0$. Therefore, the second term in Eq. (SI.41) vanishes.

The other possibility is in the event of a sufficiently strong inhibitory spike: as a consequence, the voltage can decrease immediately and the time of maximal voltage is identical to the time of this inhibitory input $\tilde{t} = t_{\tilde{\imath}+1}$. In this case, the second term will be nonzero but can be calculated because both $\dot{u}$ and $\frac{\partial \tilde{t}}{\partial \theta} = \frac{\partial t_{\tilde{\imath}+1}}{\partial \theta}$ of the inhibitory input spike $t_{\tilde{\imath}+1}$ are known. This contribution is proportional to $\dot{u}$ (the left derivative at time of input spike, i.e., how much the membrane changes in free dynamics) as well as Δt (how much a change in parameter θ influences the relevant input spike time $t_{\tilde{\imath}+1}$).

Now, we investigate $\frac{\partial u(t)}{\partial \ldots}\Big|_{t=\tilde{t}}$ in two different settings, starting with the more peculiar one.

Equal time constants $\tau_\mathrm{s} = \tau_\mathrm{m}$ In this regime the voltage behaves as

$$u(t) = \sum_i \Theta(t - t_i) \frac{w_i}{g_\ell} \frac{t - t_i}{\tau_\mathrm{s}} \exp\left(-\frac{t - t_i}{\tau_\mathrm{s}}\right) \,. \tag{SI.42}$$

We can calculate the derivative to be

$$\frac{\partial u(t)}{\partial w_j}\Big|_{t=\tilde{t}} = \frac{1}{g_\ell} \sum_{i \in \{i | t_i < \tilde{t}\}} \underbrace{\frac{\partial w_i}{\partial w_j}}_{\delta_{ij}} \frac{\tilde{t} - t_i}{\tau_\mathrm{s}} \exp\left(-\frac{\tilde{t} - t_i}{\tau_\mathrm{s}}\right) \tag{SI.43}$$

$$= \frac{1}{g_\ell} \mathbb{1}_{t_j < \tilde{t}} \frac{\tilde{t} - t_j}{\tau_\mathrm{s}} \exp\left(-\frac{\tilde{t} - t_j}{\tau_\mathrm{s}}\right) \,. \tag{SI.44}$$

Similarly, we get

$$\frac{\partial u(t)}{\partial t_j}\Big|_{t=\tilde{t}} = \frac{w_j}{g_\ell} \mathbb{1}_{t_j < \tilde{t}} \frac{\tilde{t} - t_j - \tau_\mathrm{s}}{\tau_\mathrm{s}^2} \exp\left(-\frac{\tilde{t} - t_j}{\tau_\mathrm{s}}\right) \,. \tag{SI.45}$$

Unmatched time constants $\tau_\mathrm{s} \neq \tau_\mathrm{m}$ While the voltage dynamics is slightly different

$$u(t) = \sum_i \Theta(t - t_i) \frac{w_i}{g_\ell} \frac{\tau_\mathrm{s}}{\tau_\mathrm{m} - \tau_\mathrm{s}} \left[\exp\left(-\frac{t - t_i}{\tau_\mathrm{m}}\right) - \exp\left(-\frac{t - t_i}{\tau_\mathrm{s}}\right)\right] \,, \tag{SI.46}$$

the calculation is similar and results in

$$\frac{\partial u(t)}{\partial w_j}\Big|_{t=\tilde{t}} = \frac{\tau_\mathrm{s}}{\tau_\mathrm{m} - \tau_\mathrm{s}} \frac{1}{g_\ell} \mathbb{1}_{t_j < \tilde{t}} \left[\exp\left(-\frac{\tilde{t} - t_j}{\tau_\mathrm{m}}\right) - \exp\left(-\frac{\tilde{t} - t_j}{\tau_\mathrm{s}}\right)\right] \tag{SI.47}$$

$$\frac{\partial u(t)}{\partial t_j}\Big|_{t=\tilde{t}} = \frac{\tau_\mathrm{s}}{\tau_\mathrm{m} - \tau_\mathrm{s}} \frac{w_j}{g_\ell} \mathbb{1}_{t_j < \tilde{t}} \left[\frac{1}{\tau_\mathrm{m}} \cdot \exp\left(-\frac{\tilde{t} - t_j}{\tau_\mathrm{m}}\right) - \frac{1}{\tau_\mathrm{s}} \cdot \exp\left(-\frac{\tilde{t} - t_j}{\tau_\mathrm{s}}\right)\right] \,. \tag{SI.48}$$

SI.F Relationship of weight and delay for LIF-based parrot neuron

With the LIF dynamics from above (Eq. (SI.46)), we can proceed to get the desired relationship of a weight and the resulting delay. To calculate the delay of a parrot neuron that has one input spike time t associated with a weight w, one needs to compute its time of spiking (i.e., $u(T) = \vartheta$). Assuming w.l.o.g. $t = 0$ and using $T = t + d$ for a delay d yields

$$\vartheta = \frac{\tau_\mathrm{s}}{g_\ell(\tau_\mathrm{m} - \tau_\mathrm{s})} w \left[\exp\left(-\frac{d}{\tau_\mathrm{m}}\right) - \exp\left(-\frac{d}{\tau_\mathrm{s}}\right)\right] \,. \tag{SI.49}$$

Solving for the weight w returns

$$w = \frac{g_\ell \vartheta(\tau_\mathrm{m} - \tau_\mathrm{s})}{\tau_\mathrm{s}} \frac{1}{\exp\left(-\frac{d}{\tau_\mathrm{m}}\right) - \exp\left(-\frac{d}{\tau_\mathrm{s}}\right)} \; . \tag{SI.50}$$

However, this holds only if the neuron is in fact spiking. This can happen in the interval $[0; \tilde{t}]$ with $\tilde{t}$ the time at which the membrane voltage is maximal:

$$\tilde{t} = \frac{\tau_\mathrm{m}\tau_\mathrm{s}}{\tau_\mathrm{m} - \tau_\mathrm{s}} \log \frac{\tau_\mathrm{m}}{\tau_\mathrm{s}} \; . \tag{SI.51}$$

For the specific case of $\tau_\mathrm{s} = \tau_\mathrm{m}$, l'Hôpital's rule in the limit $\tau_\mathrm{m} \to \tau_\mathrm{s}$ can be applied to Eq. (SI.50) and Eq. (SI.51):

$$w = \frac{g_\ell \vartheta \tau_\mathrm{s}}{d} \exp\left(\frac{d}{\tau_\mathrm{s}}\right) \quad \text{and} \quad \tilde{t} = \tau_\mathrm{s} \; . \tag{SI.52}$$

SI.G Simulation parameters

Table SI.2: **Dataset and training parameters**. Used to produce the results in Fig. 4, Fig. 5, Fig. SI.1, Fig. SI.2, Fig. SI.3, Fig. SI.4, Fig. SI.5 and Table SI.1.

**parameter name**	**ideal simulation**	**hardware-aware simulation/ hardware emulation**
**dataset parameters**		
input size	4	4
t_{early}	0.15	0.15
t_{late}	2.0	2.0
**training parameters**		
training epochs	300	300
batch size	150	40
adam parameter β	$(0.9, 0.999)$	$(0.9, 0.999)$
adam parameter ϵ	10^{-8}	10^{-8}
lr-scheduler	StepLR	StepLR
lr-scheduler step size	20	20
lr-scheduler γ	0.95	0.95
delay-lr[1]	$[0.1, 0.3, 0.5, 1, 1.5, 2] \times 10^{-2}$	2×10^{-3}
weight-lr[1]	$[0.1, 0.3, 0.5, 1, 1.5, 2] \times 10^{-2}$	2×10^{-3}
input noise σ	no noise	no noise
max allowed Δw	0.2	0.2
weight bump value	0.0005	0.0005
loss Δ_t	0.2	0.3

[1] For hyperparameter optimization, a grid search was performed over the range of values in brackets.

Table SI.3: **Network parameters.** Used to produce the results in Fig. 4, Fig. 5, Fig. SI.1, Fig. SI.2, Fig. SI.3, Fig. SI.4, Fig. SI.5 and Table SI.1.

parameter name	ideal simulation	hardware-aware simulation/ hardware emulation
**neuron parameters**		
g_ℓ	0.5	1.0
E_ℓ	0.0	0.0
ϑ	1.0	2.6
τ_m	2.0	1.0
τ_s	1.0	1.0
**network parameters**		
layer 0[1]	[*broadcast, axonal, dendritic, synaptic*]	
delay init mean	0.0	0.0
delay init std	0.25	0.5
scale λ	1.0	1.5
shift	0.0	2.0
layer 1	*neuron*	
size[1]	[5, 10, 15, 20, 25, 30]	
max ratio missing spikes	0.3	0.05
weight init mean	1.0	1.0
weight init std	1.0	0.12
layer 2[1]	[*broadcast, axonal, dendritic, synaptic*]	
delay init mean	0.0	0.0
delay init std	0.25	0.5
scale λ	1.0	1.5
shift	0.0	2.0
layer 3	*neuron*	
size	3	3
max ratio missing spikes	0.0	0.05
weight init mean	1.0	0.075
weight init std	1.0	0.15

[1] Parameters over which a sweep was performed are in brackets.

Table SI.4: **Weight and delay configurations.** Used to produce the results in Fig. SI.2. These values are extracted from trained networks and averaged from 10 different seeds. They were not trained after the initialization, but rather fixed. The rest of the parameters were kept the same as in Table SI.3.

**parameter name**	**ablation study**			
layer 0	*broadcast*	*axonal*	*dendritic*	*synaptic*
delay mean		0.0	0.0	0.0
delay std		0.50	1.13	0.92
layer 1	*neuron*			
weight mean	0.68	0.87	0.91	0.90
weight std	1.14	1.72	1.47	1.17
layer 2	*broadcast*	*axonal*	*dendritic*	*synaptic*
delay mean		0.0	0.0	0.0
delay std		0.85	0.21	0.75
layer 3	*neuron*			
weight mean	0.56	1.71	1.64	1.26
weight std	1.53	4.55	3.88	2.12

Table SI.5: **Specific delay configurations.** Used to produce the results in Fig. SI.3. Those values were not trained after the initialization, but rather fixed. The rest of the parameters were kept the same as in Table SI.3.

**parameter name**	**random but fixed delay**
Layer 0	*broadcast, axonal, dendritic, synaptic*
delay mean	0.0
delay std[1]	[0.0, 0.4375, 0.875, 1.3125, 1.75]
Layer 2	*broadcast, axonal, dendritic, synaptic*
delay mean	0.0
delay std[1]	[0.0, 0.4375, 0.875, 1.3125, 1.75]

[1] For hyperparameter optimization, a grid search was performed over the range of values in brackets.

Table SI.6: **Hardware parameters.** Used to produce the results in Fig. 5, Fig. SI.4, Fig. SI.5 and Table SI.1. These values were obtained from the study made in Section SI.A.3.

**parameter name**	**hw-aware**
weight quant. (max range)[1]	2.1
weight quant. (precision)	1/30
FP noise (mean)	0.13
FP noise (std)	0.08
trial-to-trial (std)	0.04
delay jitter	0.01

[1] To ensure sufficient drive at the input, the maximum range of the weight was multiplied by 5 for networks with a hidden layer of 5 neurons, for both hardware-aware simulation and hardware emulation.

References

1. DAgostino, S., Moro, F., *et al.* DenRAM: Neuromorphic Dendritic Architecture with RRAM for Efficient Temporal Processing with Delays. *Nature Communications* **15,** 3446 (2024).
2. Göltz, J., Kriener, L., *et al.* Fast and energy-efficient neuromorphic deep learning with first-spike times. *Nature Machine Intelligence* **3,** 823–835 (2021).
3. Mészáros, B., Knight, J. C. & Nowotny, T. Efficient Event-based Delay Learning in Spiking Neural Networks. *arXiv preprint arXiv:2501.07331* (2025).
4. Kriener, L., Göltz, J. & Petrovici, M. A. *The Yin-Yang Dataset* in *Neuro-Inspired Computational Elements Conference* (Association for Computing Machinery, Virtual Event, USA, 2022), 107–111.
5. Müller, E., Althaus, M., *et al. jaxsnn: Event-driven Gradient Estimation for Analog Neuromorphic Hardware* in *2024 Neuro Inspired Computational Elements Conference (NICE)* (2024), 1–6.
6. Göltz, J., Billaudelle, S., *et al.* Gradient-based methods for spiking physical systems. *International conference on neuromorphic, natural and physical computing (NNPC).* arXiv: `2309.10823 [q-bio.NC]` (2023).
7. Wunderlich, T. C. & Pehle, C. Event-based backpropagation can compute exact gradients for spiking neural networks. *Scientific Reports* **11** (June 2021).
8. Brette, R. & Gerstner, W. Adaptive Exponential Integrate-and-Fire Model as an Effective Description of Neuronal Activity. *Journal of Neurophysiology* **94,** 3637–3642 (Nov. 2005).
9. Pehle, C., Billaudelle, S., *et al.* The BrainScaleS-2 Accelerated Neuromorphic System with Hybrid Plasticity. *Front. Neurosci.* **16.** arXiv: `2201.11063` (2022).
10. Billaudelle, S., Weis, J., *et al. An accurate and flexible analog emulation of AdEx neuron dynamics in silicon* in *2022 29th IEEE International Conference on Electronics, Circuits and Systems (ICECS)* (2022), 1–4.
11. Perez-Nieves, N., Leung, V. C. H., *et al.* Neural heterogeneity promotes robust learning. *Nature Communications* **12** (Oct. 2021).
12. Cramer, B., Billaudelle, S., *et al.* Surrogate gradients for analog neuromorphic computing. *Proceedings of the National Academy of Sciences* **119** (2022).
13. Bittar, A. & Garner, P. N. A surrogate gradient spiking baseline for speech command recognition. *Frontiers in Neuroscience* **16,** 865897 (2022).
14. Nowotny, T., Turner, J. P. & Knight, J. C. Loss shaping enhances exact gradient learning with Eventprop in spiking neural networks. *Neuromorphic Computing and Engineering* **5,** 014001 (Jan. 2025).
